# Unveiling the dynamics of intraoperative contamination in total hip arthroplasty: the discrepancy between particulate and microbial contamination in surgical site infection risk

**DOI:** 10.1186/s42836-024-00272-0

**Published:** 2024-10-01

**Authors:** Toshiyuki Tateiwa, Toshinori Masaoka, Yasuhito Takahashi, Tsunehito Ishida, Takaaki Shishido, Kengo Yamamoto

**Affiliations:** 1 Department of Orthopedic Surgery, Tokyo Medical University, 6-7-1, Nishishinjuku, Shinjuku-ku, Tokyo, 160-0023 Japan; 2 Department of Bone and Joint Biomaterial Research, Tokyo Medical University, 6-7-1, Nishishinjuku, Shinjuku-ku, Tokyo, 160-0023 Japan

**Keywords:** Total hip arthroplasty, Surgical site infection, Space suits, Particle contamination, Microbial contamination

## Abstract

**Background:**

Surgical site infection (SSI) is a major problem following total hip arthroplasty (THA). This study investigated the impact of a standard intraoperative routine where the surgical team wears full-body exhaust suits (space suits) within a laminar airflow (LAF)-ventilated operating room (OR) on environmental contamination. Our primary objective was to identify potential modifiable intraoperative factors that could be better controlled to minimize SSI risk.

**Methods:**

We implemented an approach involving simultaneous and continuous air sampling throughout actual primary cementless THA procedures. This method concurrently monitored both airborne particle and microbial contamination levels from the time the patient entered the OR for surgery until extubation.

**Results:**

Airborne particulate and microbial contamination significantly increased during the first and second patient repositionings (postural changes) when the surgical team was not wearing space suits. However, their concentration exhibited inconsistent changes during the core surgical procedures, between incision and suturing, when the surgeons wore space suits. The microbial biosensor detected zero median microbes from draping to suturing. In contrast, the particle counter indicated a significant level of airborne particles during head resection and cup press-fitting, suggesting these procedures might generate more non-viable particles.

**Conclusions:**

This study identified a significant portion of airborne particles during the core surgical procedures as non-viable, suggesting that monitoring solely for particle counts might not suffice to estimate SSI risk. Our findings strongly support the use of space suits for surgeons to minimize intraoperative microbial contamination within LAF-ventilated ORs. Therefore, minimizing unnecessary traffic and movement of unsterile personnel is crucial. Additionally, since our data suggest increased contamination during patient repositioning, effectively controlling contamination during the first postural change plays a key role in maintaining low microbial contamination levels throughout the surgery. The use of sterile gowns during this initial maneuver might further reduce SSIs. Further research is warranted to investigate the impact of sterile attire on SSIs.

## Introduction

Deep infection following total hip arthroplasty (THA) represents a devastating complication associated with significant patient morbidity and a substantial burden on individual patients, surgeons, and the healthcare system. Reported data suggested a nationwide annual incidence of periprosthetic joint infection (PJI) following THA standing at approximately 2%–3% [1, 2]. Furthermore, the associated mortality rate has been reported to be as high as 23.6% [3]. The economic burden of THA-associated PJI is projected to increase in the United States, with estimates reaching $753.4 billion by 2030, primarily driven by the rising volume of procedures [4]. Given this context, identifying risk factors for surgical site infection (SSI) and implementing evidence-based prevention strategies are of paramount importance in the field of arthroplasty [5].

The risk factors for SSI are categorized into two major groups. The first group consists of intrinsic factors inherent in patients because of underlying medical conditions. They include age, sex, weight, medical history (e.g., diabetes, immunodeficiency), and preoperative carrier problems, among others [6, 7]. The problem lies in that it tends to be difficult or impossible for surgeons to control some of these factors. The second group involves extrinsic factors related to operating room (OR) personnel and environment. Some of these factors can potentially be eliminated by revising intraoperative routines to minimize SSI.

The intraoperative use of laminar airflow (LAF) systems and full-body exhaust suits (space suits) has been a subject of controversy [8–15]. Historically, Charnley recommended the use of LAF and body exhaust suits because they lowered the infection rate after primary THA from 9.5% to 0.5% [8, 9]. However, according to a nationwide study conducted in Germany, upon adjustment of confounding factors, multivariate analysis revealed that OR ventilation with LAF was not associated with lower infection risk [13]. Moreover, a recent systematic review concluded that the risk of severe SSI was significantly higher in THA in LAF-ventilated ORs as compared to conventional ventilation [15]. On the other hand, several recent studies reported that a space suit may have no benefit or even be associated with higher infection risk [10, 11, 14]. Therefore, the impact of LAF and space suits on SSI prevention needs further investigation, particularly regarding microbial contamination.

The level of airborne contamination in the OR environment has been considered a significant risk indicator for SSI [16–21]. Friberg et al. [19] experimentally demonstrated that airborne contamination in the wound and instrumental areas was strongly correlated with surface contamination with bacteria-carrying particles in the same areas, on the patient chest, and in the periphery of the OR. Personnel and their activities considerably affect airborne contamination in the OR [20–22]. We thus hypothesized that an intraoperative movement of personnel dressed in space suits would influence the level of airborne microbial contamination in the OR.

This study investigated the impact of a standard intraoperative routine where the surgical team wore space suits within an LAF-ventilated OR on environmental contamination. Our primary objective was to identify potential modifiable intraoperative factors that could be better controlled to minimize SSI risk.

To achieve this, we implemented an approach involving simultaneous and continuous air sampling throughout actual primary cementless THA procedures. This method concomitantly monitored both airborne particulate and microbial contamination levels from the time the patient entered the OR for surgery until extubation. This combined analysis strategy was chosen for two key reasons. First, while microbial contamination at the surgical site is a well-established risk factor for SSIs [23, 24], airborne particulate contamination may not always directly contribute to infection due to the presence of non-viable particles (e.g., dust). Second, by measuring both types of contaminants simultaneously, we aimed to gain a more comprehensive understanding of the potential interactions between airborne particles and microbial burden during THA procedures. This comprehensive dataset will ultimately inform the development and implementation of targeted preventive measures specifically designed to minimize SSI risk within the context of LAF-ventilated ORs utilizing space suits.

## Methods

This study examined the airborne particulate and microbial contamination during THA. The study focused solely on the OR environment and did not involve any patient interventions. Therefore, patient details were not collected and are not relevant to this investigation. Since this study principally compared different stages within common THA procedures, a control group wasn’t required. Notably, our institution’s standard practice mandates space suits be used during THA to mitigate concerns regarding surgical contamination and safeguard the health of both surgeons and patients.

The airborne particle concentrations and microbial reaction rates were continuously monitored during seventeen primary cementless THAs performed in the same OR with a vertical LAF (room volume: 130.75 m^3^; floor area: 44.25 m^2^), satisfying a standard for clean room of the National Aeronautics and Space Administration (NASA class 100). All surgeries were performed between May 2017 and December 2018. Four experienced hip surgeons (TT, TM, TS, and KY) performed all operations via posterior approach under general anesthesia, starting at 8:30 a.m.

**Fig. 1:**
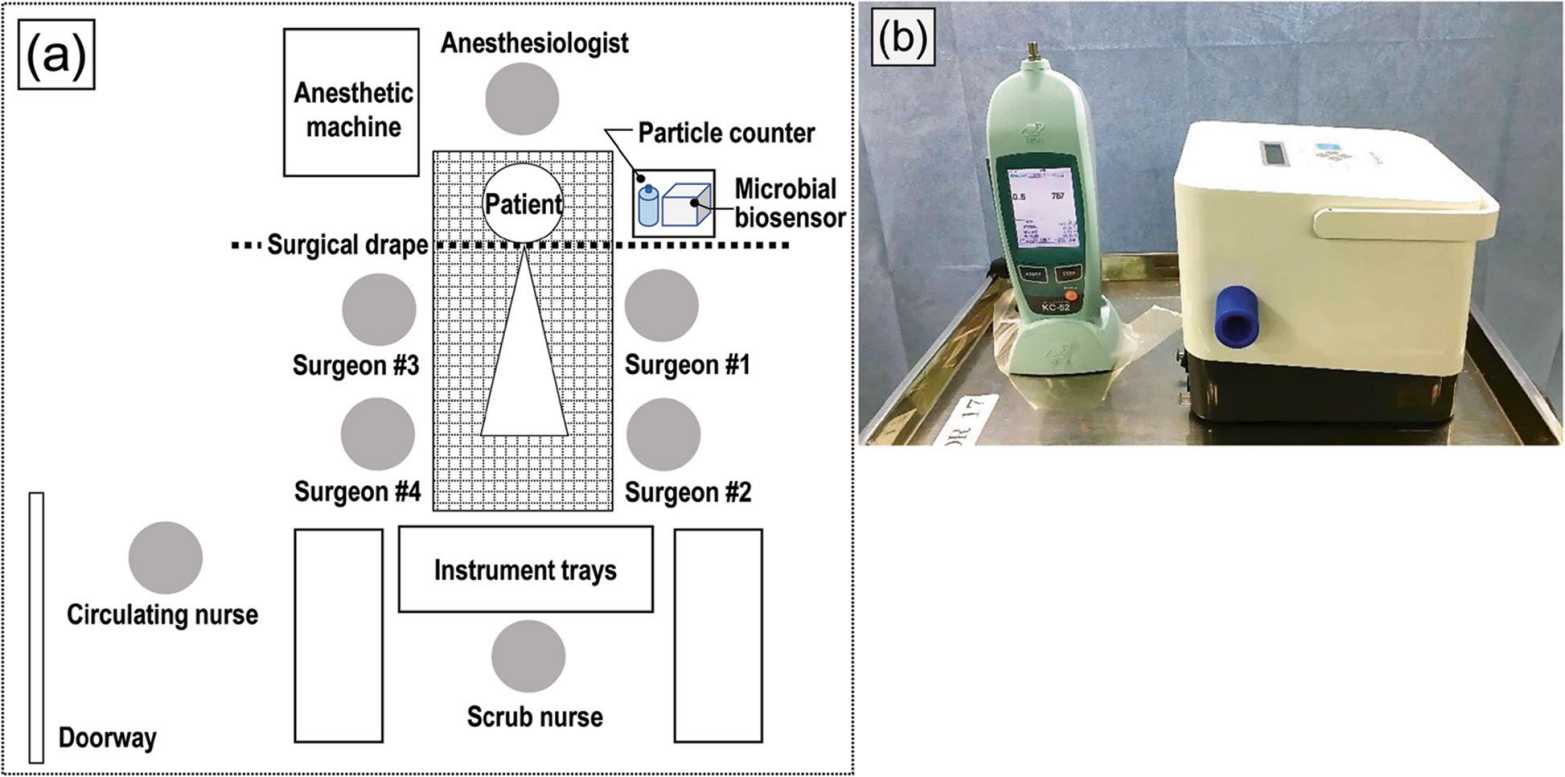
**a** A schematic illustrating the experimental setup in the LAF-ventilated OR; **b** A photograph of the particle counter and microbial biosensor used in this study

The experimental setup is illustrated in Fig. 1a. The surgical team was made up of seven members, including four surgeons, an anesthesiologist, a scrub nurse, and a circulating nurse. Particle counts and microbial reaction rates were recorded at the following surgical steps: (1) patient entry into the OR; (2) first postural change; (3) draping; (4) incision; (5) femoral head resection; (6) acetabular cup press-fitting; (7) femoral stem press-fitting; (8) suturing; (9) second postural change; and (10) extubation. Four surgeons and one scrub nurse wore sterile full-body space suits (Stryker Instruments, Kalamazoo, MI, USA) from steps (3) to (8). The OR personnel wore regular unsterile surgical scrubs for steps (1), (2), (9), and (10). Due to the need for rigorous control of access to the OR, environmental examinations were randomly conducted within the specified period. We consistently maintained the same surgical staff of seven, and restricted entry/exit from the room over the duration of the study.

The airborne particle levels were measured using a hand-held particle counter (KC-52, Rion Co., LTD., Tokyo, Japan) (Fig. 1b). Particles were counted one by one using a photodiode detector, with coincidental loss occurring when two particles passed through the sensor simultaneously. With the counter used, coincidental loss was within 10% of the indicated readings at a count of 1.4 × 10^5^ particles per cubic meter. Airborne particles with a size of ≥ 0.3 μm (roughly corresponding to the diameter of squamous epithelial cells [18]) were counted within a volume of ≈2.83 L around the detector for 1 min. A hand-held particle-counter was set up at a height of 1 m from the floor, 1 m to the left of the patient’s head area.

A microbial biosensor (BM-300C; Sharp Life Science Co. Ltd., Hyogo, Japan) deployed adjacent to the particle counter (Fig. 1b), measured the microbial reaction rate per 1 m^3^ in measurement cycles involving 5 min of continuous suction followed by 5 min of continuous testing. A separation of microbial and non-microbial particles less than 10 μm was achieved, and the fine particles electrostatically collected on a collection plate were heated at 200 °C for 1 min to promote the Maillard reaction. After irradiation with excitation light (laser diode: 405 nm), the fluorescence emission intensity was measured and is directly proportional to the microbial concentration. Therefore, a high microbial reaction rate indicates an active and abundant microbial population and can be used as an indirect indicator of a high microbial concentration.

All results were statistically analyzed using the non-parametric Kruskal-Wallis test followed by Dunn’s post hoc test performed with the aid of GraphPad Prism software, version 8.3.0 (GraphPad Software, Inc., San Diego, CA, USA). The differences were considered to be statistically significant at the *P* < 0.05 level.

## Results

Table 1 shows the time (mean and standard deviation [SD]) time from the first postural change to extubation in THA procedures.

**Table 1:** The mean time elapsed from patients’ entry into the OR in the primary cementless THAs

**Surgical steps**	**Time [min]**	
	**Mean**	**SD**
Patient entry into the OR	0	0
First postural change	23	6
Draping	47	7
Incision	56	8
Head resection	65	8
Cup press-fitting	83	12
Stem press-fitting	104	15
Suturing	120	17
Second postural change	126	16
Extubation	148	24

**Fig. 2:**
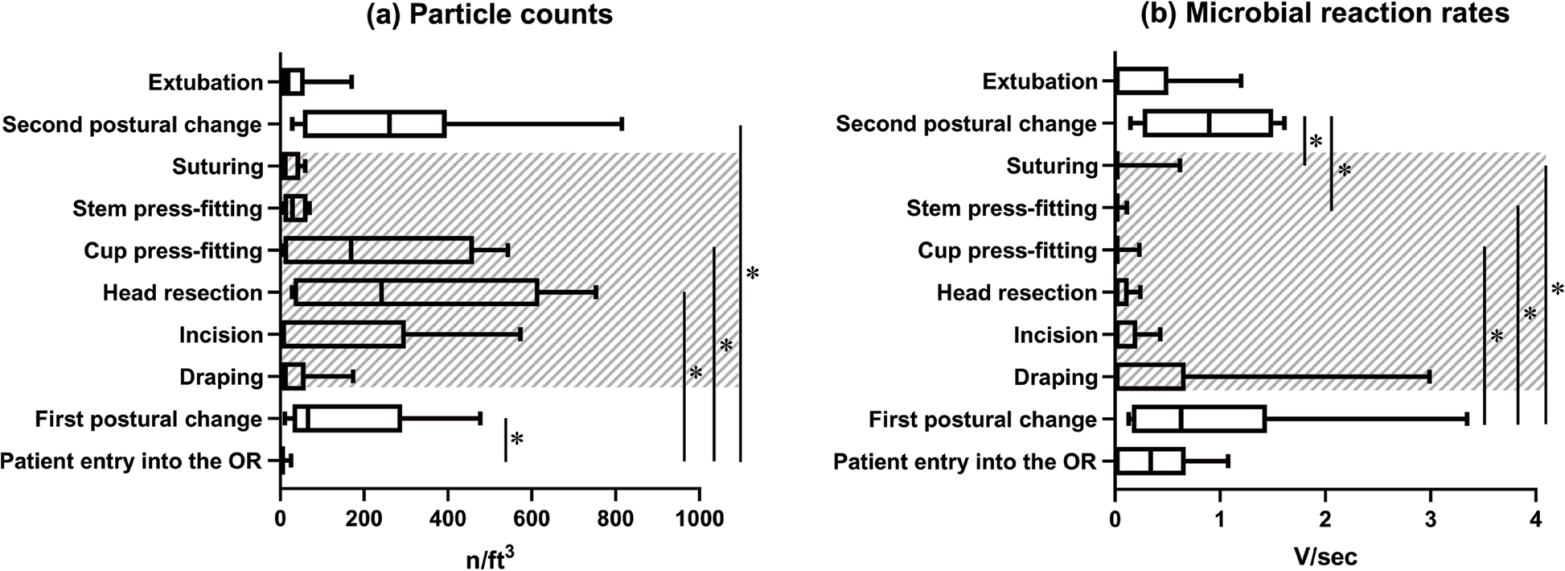
Boxplot diagrams of airborne particle counts (**a**) and microbial reaction rates (**b**) in the OR environment in response to intraoperative routine procedures of the surgical team in primary cementless THAs. The shaded areas indicate the period when the four surgeons and one scrub nurse were dressed in full-body space suits. Asterisks represent *P* < 0.05 for Dunn’s post hoc test following the significant Kruskal-Wallis test

The median (interquartile, IQR) intraoperative particle counts (n/ft^3^) in each surgical step were as follows: 1 (0–6) at patient entry into the OR, 66 (48–213) at the first postural change, 13 (4–41) for draping, 8 (4–77) at the incision, 242 (35–492) at head resection; 169 (11–365) at cup press-fitting, 29 (10–56) at stem press-fitting, 12 (4–45) at suturing, 260 (64–377) at the second postural change, and 19 (4–50) at extubation (Fig. 2a). The lowest median particle concentration was observed at patient entry into the OR, whereas the peak median concentration was at the second postural change. The Kruskal-Wallis test revealed that the airborne particle concentration in the OR significantly changed in response to surgical steps (*P* < 0.0001). According to Dunn’s post hoc tests, significantly higher concentrations were observed at the first postural change (*P* = 0.004), head resection (*P* = 0.001), cup press-fitting (*P* = 0.039), and the second postural change (*P* = 0.0003) as compared to patient entry into the OR.

The median (IQR) intraoperative microbial reaction rate (V/sec) in each surgical step was as follows: 0.3 (0.0–0.6) at patient entry into the OR, 0.6 (0.3–0.8) at the first postural change, 0.0 (0.0–0.4) for draping, 0.0 (0.0–0.2) at the incision, 0.0 (0.0–0.1) at head resection, 0.0 (0.0–0.0) at cup press-fitting, 0.0 (0.0–0.0) at stem press-fitting, 0.0 (0.0–0.0) at suturing, 0.9 (0.5–1.3) at the second postural change, and 0.0 (0.0–0.3) at extubation, (Fig. 2b). The lowest median microbial concentrations were observed from draping to suturing and at extubation, whereas the peak median concentration was found at the second postural change. The Kruskal-Wallis test revealed that the airborne microbial concentration in the OR significantly changed in response to surgical steps (*P* = 0.0003). Dunn’s post hoc tests showed significant differences between the first postural change and cup press-fitting (*P* = 0.037), the first postural change and stem press-fitting (*P* = 0.010), the first postural change and suturing (*P* = 0.019), the second postural change and stem press-fitting (*P* = 0.025), and the second postural change vs. suturing (*P* = 0.042).

## Discussion

This study investigated the fluctuations of airborne particulate and microbial concentrations within an LAF-ventilated OR during routine THA procedures. As compared to the patient’s entry into the OR, a significant increase in airborne particles was found at the first postural change, head resection, cup press-fitting, and the second postural change (Fig. 2a). On the other hand, a significant increase in airborne microbes was noted at the first and second postural change as compared to implant insertion and suturing (Fig. 2b). An interesting finding was the inconsistency between airborne particulate and microbial contamination during the core surgical steps from incision to suturing when the surgeons wore space suits (see the shaded areas in Fig. 2). The particle counter showed a significant level of intraoperative airborne particles at the head resection and cup press-fitting. In contrast, the microbial biosensor detected zero median microbes from draping to suturing. These results indicated that the intraoperative airborne particles might be predominantly non-viable.

Nevertheless, recent large-scale clinical studies questioned the rationale for the routine use of space suits and LAF in THA because their use is likely to provide no increased protection against bacterial contamination and, quite on the contrary, is a risk factor for SSI [11–15]. A space suit creates positive pressure with air constantly flowing out, which may increase the risk of wound contamination or infection by circulating contaminants through the OR via air currents [20, 25]. Besides, intraoperative personnel movements as well as the air flows from laminar systems may facilitate the contamination process. In the present study, the increased airborne particles indeed took place while wearing space suits. Because the contamination level is directly correlated with the activity level of OR personnel [22], the significantly higher particle counts at head resection and cup press-fitting are likely associated with the surgeons’ relatively active movements. However, this non-microbial contamination is unlikely to be a significant risk factor for SSI. We speculate that bone dust emitted from the patient and/or clean microfibers from sterile space suits or drapes could be possible sources of the increased particles. In the aforementioned context, our hypothesis was not supported by the present findings, and bacteria were not shed intraoperatively owing to the use of enclosed hoods and exhaust systems combined with occlusive gowns even under the condition of air flows from laminar systems. Hooper et al. [14], however, cautioned the possibility of unknowing contamination of the gloves through frequent intraoperative adjustment of the suit or hood.

The most important finding was that significantly increased levels of both airborne particles and microbes were found during patient repositionings when the surgical team was not wearing space suits. The results suggest a strong association between microbial fallout and the movement of OR personnel in unsterile attire. In a previous experimental study, considerable airborne contamination (beyond the level of NASA100 threshold) was found when a surgical member used an unsterile scrub uniform or unsterile outside shoes [21]. Therefore, we recommend that the traffic of personnel from outside dressed in unsterile attire into the OR should be regulated. Additionally, minimizing the size of the surgical team is advisable. Several studies have documented that opening the OR door disrupted LAF, allowing pathogens to enter the space surrounding the surgical site [26–29]. To mitigate this risk, personnel from outside the OR should wear sterile surgical gowns and minimize their activity level inside the OR, especially during the critical steps between draping and suturing.

Our findings strongly suggest that wearing space suits effectively reduces microbial contamination during surgery (Fig. 2b). This is likely because sterile space suits completely cover unsterile surgical attire, acting as a physical barrier that suppresses the release of microbes from the surgeon to the surgical field. Notably, studies have shown that microbial contamination tends to occur during patient repositioning when wearing unsterile attire. Subsequently, it takes approximately one hour, from the time of the first postural change to the time of cup press-fitting, for contamination levels to significantly decrease. Therefore, effectively suppressing microbial contamination during the first postural change is crucial for maintaining low contamination levels throughout the surgery. Conversely, the observed increase in contamination during the second postural change is unlikely to impact SSI as it occurs after surgical wound closure.

This study has limitations. First, since there is a correlation between the number of people present in the OR and microbial counts [16, 30], we consistently maintained the same surgical staff of seven, and restricted entry/exit from the room during the study. However, in daily clinical practice, there is random traffic of additional unsterile personnel entering and exiting the OR with associated door opening and closing. This could potentially lead to greater contamination compared to the results presented here. Second, the detected increased level of airborne microbes may not lead directly to SSI. However, it was previously reported by a Medical Research Council that there was a correlation between contamination levels in the air and the frequency of postoperative SSI [24].

## Conclusions

This study identified a significant portion of airborne particles during the core surgical steps as non-viable, suggesting that monitoring solely for particle counts might not be sufficient to estimate SSI risk. Our findings strongly support the use of space suits for surgeons to minimize intraoperative microbial contamination within LAF-ventilated ORs. Therefore, minimizing unnecessary traffic and the movement of unsterile personnel is crucial. Additionally, as our data suggest increased contamination during patient repositioning, effectively controlling contamination during the first postural change plays a key role in maintaining low microbial contamination levels throughout the surgery. The use of sterile gowns during this initial maneuver might further reduce SSIs. Further research is warranted to better understand the impact of sterile attire on SSIs.

## Data Availability

All data analyzed during this study are included in this article.
